# A hindered phenol containing PVC/CuO nanocomposites; study on the mechanical and thermooxidative properties

**DOI:** 10.55730/1300-0527.3708

**Published:** 2024-12-27

**Authors:** Mohsen HAJIBEYGI, Alireza GHASEMI

**Affiliations:** Department of Organic and Polymer Chemistry, Faculty of Chemistry, Kharazmi University, Tehran, Iran

**Keywords:** Poly(vinyl chloride), nanocomposite, CuO nanoparticles, hindered phenol, thermooxidation

## Abstract

The effect of synthesized 5-((4-hydroxy-3,5-di-tert-butylphenyl)diazenyl)isophthalic acid (HBA) containing a hindered phenol derivative on the thermooxidation, hydrochloric acid release time, and mechanical strength of PVC/CuO nanocomposites was studied. Moreover, 5-((4-hydroxy-2,5-dimethylphenyl)diazenyl)isophthalic acid (HMA) was synthesized for comparison of corresponding PVC nanocomposite properties. PVC nanocomposite thin films were prepared through in situ surface modification of CuO nanoparticles with HBA and HMA, individually, in the PVC solution. The XRD and FE-SEM results clarified the desirable dispersion of CuO nanoparticles. The PVC sample with loading of 5 wt% from each HBA and CuO was found to be the most thermally stable, which was confirmed by thermogravimetric analysis in inert conditions. The thermooxidation and Congo red test results revealed that the simultaneous loading of HBA and CuO nanoparticles into the PVC matrix could increase the initial thermal degradation and the stability times. Moreover, the PVC sample containing 2.5 wt% each from HBA and CuO nanoparticles exhibited tensile strength almost 20 MPa more than that of neat PVC.

## 1. Introduction

Poly(vinyl chloride) (PVC), because of its beneficial properties such as a relatively high glass transition temperature and being economical and durable, is one of the most widely used polymeric materials in various applications, in comparison to other commercial polymers. PVC products are used in short- and long-term applications, such as various car parts, construction, floor coverings, cable insulation, profiles, and hoses [1]. However, PVC is also sensitive to heat, photooxidation, and thermooxidation during use and the processing steps, compared to polystyrene, polyethylene, and polypropylene, other commercial thermoplastics [2]. Thermal decomposition of PVC, which is accompanied by an autocatalytic reaction and HCl elimination simultaneously, forms a polymeric structure with a conjugated double bond. This process changes the color of the polymer and causes drastic damage to its mechanical, physical, and chemical properties [3,4].

Generally, the loading of inorganic nanomaterials along with effective antioxidants into polymer matrices could reduce the thermal degradation and degeneration of the main properties during processing and use. During this process, various desirable properties like electrical and mechanical properties, heat resistance, and fire retardancy are also improved [2,5–8].

The loading of inorganic nanoparticles into polymers with poor properties has provided promising performance such as in catalytic and antibacterial activity and development of improved materials with desirable electrical and mechanical properties, flame retardancy, and thermal stability [9]. Inorganic nanomaterial fillers play an influential role in attaining favorable properties and decreasing the cost of the nanocomposites produced [10]. Transition metal oxide nanoparticles have unique physicochemical properties originating from their high surface area, which may not be the same as that of their bulk or atomic counterparts [11]. Copper oxide (CuO), a p-type metal oxide semiconductor, features a narrow bandgap that contributes to its unique properties. It has been widely utilized in various applications, including optical and electronic devices, photovoltaic systems, solar energy harvesting, catalysis, gas sensors, and magnetic storage technologies [12,13]. The thermal degradation of rigid PVC has been studied by the loading of unmodified CuO and the results exhibited better thermal stability in the resulting nanocomposites compared to neat PVC [14]. Furthermore, loading CuO into the PVC matrix can decrease its pyrolysis temperature and reduce the formation of toxic substances [10]. However, the aggregation of nanoparticles and their poor compatibility with the polymer matrix remain significant challenges in fabricating nanocomposites. These issues can be addressed through strategies such as premodification of nanoparticles before incorporation into the polymer, in situ surface modification during nanocomposite synthesis, or the simultaneous application of coupling agents [15–18]. The dispersion of CuO nanoparticles was investigated by Jeng et al. [15] using a modification of the oleate structure and by Ghashghaee et al. [19] using stearic acid as a surface modifier for uniform dispersion in the polymer matrix.

Antioxidants containing free radical scavenger groups have been promoted to prevent and decrease oxidative processes and accordingly can increase the lifetime of materials, cure diseases and inflammation, and delay the aging process [20,21]. Antioxidants containing sterically hindered phenols, which have been introduced as materials with free radical scavenging capabilities, are the most commonly used class of antioxidants [22] and have been broadly used as a thermal stabilizer for polymers during processing and use. It is worth noting that most of the available antioxidants, and other polymer stabilizers and additives as well, have low molecular weight; therefore, they are sensitive to leaching and migration [23]. In order to reduce the leaching, migration, and physical loss of polymer stabilizers and antioxidants, various intensive research has been reported and, for this, high molecular weight antioxidants were prepared and developed [24–27]. Another possible route is to immobilize antioxidant compounds in the polymer matrix by the addition of inorganic nanomaterials and the preparation of a polymer nanocomposite [28,29]. It not only can obviously improve the leaching and migration resistance of antioxidants in the polymer matrix but also increase the reinforcing effect of nanomaterial for the final resulting polymer nanocomposite [25].

In the present work, for decreasing HCl release from the PVC backbone and its catalytic effect on thermal decomposition of PVC, CuO nanoparticles were used for the preparation of PVC/CuO nanocomposites. Furthermore, according to the scavenging free radical effect of the organic hindered phenol derivatives during thermooxidation of PVC, a hindered phenol derivative was synthesized and used as reinforcement of the PVC/CuO nanocomposites. In comparison to prior studies [18], which employed thiamine as a biosafe modifier to improve the compatibility of CuO nanoparticles with the PVC matrix, our research introduces hindered phenol derivatives as surface modifiers. The hindered phenol derivatives contribute additional functionalities, including radical scavenging capabilities. These functionalities not only mitigate nanoparticle aggregation but also actively suppress the catalytic effects of hydrochloric acid on PVC degradation.

Based on the authors’ information, other studies in this field have utilized unmodified copper oxide to enhance the properties of PVC polymer blends. Ayaz et al. described thermal resistance of PVC/PMMA/CuO nanocomposites [30] and Elbasiony et al. reported CuO loading in the PVC/PE matrix [31]. In some other studies, CuO has been used in combination with other nanostructure materials, such as MgO, carbon nanotubes, and Al nanoplates to improve various properties of PVC [32–34]. Herein, the dual functionality of hindered phenol as antioxidants and surface modifiers introduces a synergistic effect, enhancing not only thermal and mechanical properties but also delaying hydrochloric acid release. This approach addresses the challenges of nanoparticle aggregation and poor compatibility reported in earlier works. Moreover, the proposed mechanism underlying these improvements is extensively explored, distinguishing this study from the existing literature.

## 2. Experimental

### 2.1. Materials

The following materials were utilized in the study: PVC: S-6558 grade, supplied by Bandar Emam (Basparan) Company, Iran; copper(II) nitrate trihydrate (Cu(NO_3_)_2_·3H_2_O): used as a precursor for CuO nanoparticle synthesis and purchased from Sigma-Aldrich; 5-aminoisophthalic acid (AIA): base material for synthesizing dicarboxylic acids and obtained from Sigma-Aldrich; 2,5-dimethylphenol and 2,6-di-tert-butylphenol: phenolic derivatives used for the synthesis of HMA and HBA and procured from Sigma-Aldrich; sodium nitrite (NaNO_2_): used in the diazonium coupling reaction and purchased from Merck; hydrochloric acid (37%) and sodium hydroxide: analytical-grade reagents used for pH adjustments and sourced from Merck; tetrahydrofuran (THF): solvent for nanocomposite preparation, analytical grade, and supplied by Merck; and deionized (DI) water: used in the preparation and purification steps.

### 2.2. Equipment

The FTIR spectra were recorded with a PerkinElmer RXI spectrometer. A Bruker instrument 300 MHz NMR instrument (Germany) and a PW1730 diffractometer (Philips, Netherlands) were used for recording the ^1^H and ^13^C NMR spectra and XRD patterns, respectively. The cross-section morphology of the sample films was observed by a MIRA3, TESCAN-XMU, field-emission scanning electron microscope (FE-SEM). The heat degradation behavior of the samples was recorded in two different conditions by a thermogravimetric analysis (TGA) instrument (Q600, TA Instruments, USA). For the T_g_ recording, differential scanning calorimetry (DSC) was used with a Q600, in argon atmosphere and with a 10 °C/min heating rate. The Congo red test was carried out according to ISO 182-1: 1990. The strength stress–strain curves were provided by a Testometric stm50 tensile test (SANTAM), with a traction rate of 5 mm/min.

### 2.3. Synthesis of the phenol-containing dicarboxylic acids

Two dicarboxylic acids, namely 5-((4-hydroxy-2,5-dimethylphenyl)diazenyl)isophthalic acid (HMA) and 5-((4-hydroxy-3,5-di-tert-butylphenyl)diazenyl)isophthalic acid (HBA), were synthesized following the procedures described previously [35]. For preparation of HMA, first the diazonium salt was synthesized as follows: 0.25 g (1.35 mmol) of 5-aminoisophthalic acid, 10 mL of water, and 0.5 g of Na_2_CO_3_ were placed in a 50-mL flask and stirred at 5 °C. A solution including 0.225 g of NaNO_2_ and 2 mL of water was added to the 5-aminoisophthalic acid solution. Subsequently, the prepared mixture was introduced into a solution containing 2.5 mL of 37% hydrochloric acid and 10 mL of water, maintained at 4 °C. After that, 0.1685 g (1.35 mmol) of 2,5-dimethyl phenol was dissolved in 10 mL of NaOH and Na_2_CO_3_ (1:1) (10%) solution in an ice bath. Finally, the diazonium salt solution was added to the phenol solution dropwise at 0 °C. The solution was stirred at 10 °C for 15 min and 25 °C for 30 min and the product was filtered, washed with water, and dried at 40 °C.

**HMA**: yield: 94%, mp: 280 °C. FT-IR (KBr): 2200–3600 (m, br), 3482 (m), 3087 (m, sh), 2923 (m, sh), 1703 (s), 1611 (s), 1585 (s), 1472 (s), 1417 (s), 1287 (s), 1082 (s), 918 (s) cm^−1^. ^1^H NMR (DMSO-d_6_, TMS) δ: 13.49 (s, 2H), 10.29 (s, 1H), 8.51 (t, 1H), 8.47 (d, 2H), 7.55 (s, 1H), 6.80 (s, 1H), 2.56 (s, 3H), 2.13 (s, 3H) ppm. ^1^H NMR (DMSO-d_6_, D_2_O exchange, TMS) δ: 8.45 (s, 1H), 8.39 (s, 2H), 7.47 (s, 1H), 6.76 (s, 1H), 2.49 (s, 3H), 2.08 (s, 3H) ppm. ^13^C NMR (DMSO-d_6_, TMS) δ: 166.83, 150.45, 153.28, 143.44, 140.06, 132.85, 131.25, 127.02, 123.83, 115.49, 116.95, 17.38, 16.11 ppm.

**HBA**: yield: 76%, mp > 300 °C, FT-IR (KBr): 2300–3700 (s, br), 3621 (m), 3322 (s), 3104 (m), 2957 (s), 2916 (m, sh), 1733 (s, sh), 1696 (s), 1610 (s), 1417 (s), 1363 (s), 1176 (s), 1008 (s) 920 (s) cm^−1^. ^1^H NMR (DMSO-d_6_, TMS) δ: 13.33 (s, 2H), 11.64 (s, 1H), 8.19 (s, 2H), 8.09 (s, 1H), 7.72 (s, 1H), 7.06 (s, 1H), 1.26 (s, 9H), 1.22 (s, 9H) ppm. ^1^H NMR (DMSO-d_6_, D_2_O exchange, TMS) δ: 8.16 (s, 2H), 8.07 (s, 1H), 7.68 (s, 1H), 7.04 (s, 1H), 1.26 (s, 9H), 1.24 (s, 9H) ppm. ^13^C NMR (DMSO-d_6_, TMS) δ: 185.61, 166.47, 149.52, 145.75, 143.96, 136.58, 132.33, 118.93, 117.45, 35.80, 34.53, 30.01 ppm.

### 2.4. Preparation of CuO nanoparticles

In a 100-mL beaker, 4.8 g of Cu(NO_3_)_2_.3H_2_O and 20 mL of DI H_2_O were placed and stirred until the formation of a homogeneous solution and a solution of sodium hydroxide (10%) was added to the copper solution with pH adjusted to around 9. The blue solid product was filtered, washed with deionized water, dried at 80 °C, and subsequently calcined at 500 °C for 4 h in a furnace, resulting in the formation of brown CuO nanoparticles. FT-IR (KBr): 3401 (m, br), 1733 (w), 1628 (w), 1499 (w), 1115 (m), 594 (s), and 503 (s) cm^−1^.

### 2.5. Preparation of the PVC nanocomposites

Two series of PVC/CuO nanocomposites were prepared containing HMA and HBA by solvent casting. For this purpose, a suspension of CuO in THF was prepared and suitable amounts of HMA or HBA were added to the PVC solution in THF. The wt% content of each component in the PVC samples is provided in Table 1. To prepare the PNMA10 sample, 5 wt% CuO nanoparticles, 5 wt% HMA, and 20 mL of THF were mixed and sonicated for 45 min. The CuO suspension was added to 30 mL of a THF solution containing 90 wt% PVC and stirred for 20 h at 25 °C, with intermittent sonication. Finally, the PNMA10 thin film was prepared by casting the mixture into a petri dish and allowing the solvent to evaporate at room temperature.

## 3. Results and discussion

### 3.1. Characterization of dicarboxylic acids

**Scheme 1:**
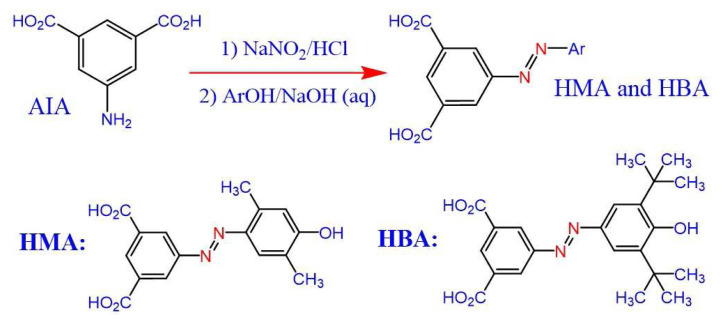
The preparation routes for HMA and HBA.

Two dicarboxylic acids containing phenol derivatives and azo functional groups were prepared from 5-aminoisophthalic acid and two derived phenols by an azo coupling reaction (Scheme 1).

**Figure 1:**
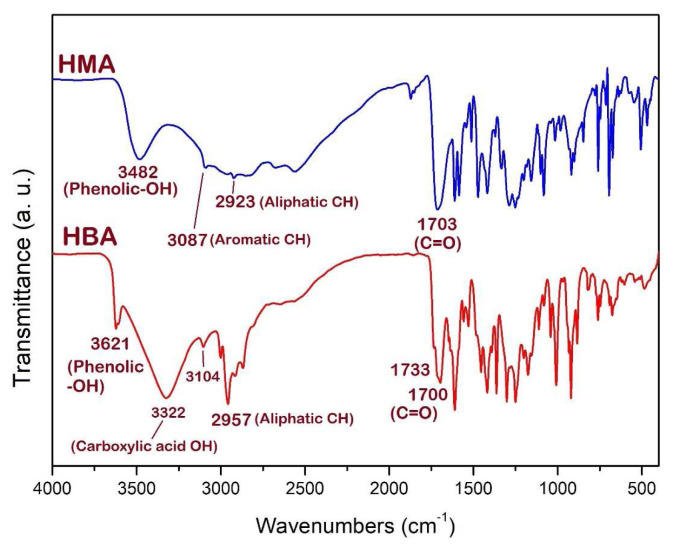
FTIR spectra of HMA and HBA.

The chemical structures of HMA and HBA were studied by FTIR and NMR spectroscopy. The FTIR spectra of HMA and HBA are shown in Figure 1. In both FTIR spectra, the absorption peak at around 2400–3500 cm^−1^ is attributed to the stretching mode of OH in the carboxylic acid groups, which appeared broad due to H-bonding generation. The carbonyl groups are observed at around 1700 cm^−1^, which is caused by its stretching mode. The peak at 1733 cm^−1^ in the FTIR spectrum of HBA may be related to the free carbonyl in its tautomeric structure [36]. Further, due to the lack of H-bonding in the OH group in HBA, its absorption band appeared at 3621 cm^−1^, while in the HMA spectrum, it appeared as a broader band at 3482 cm^−1^.

**Figure 2:**
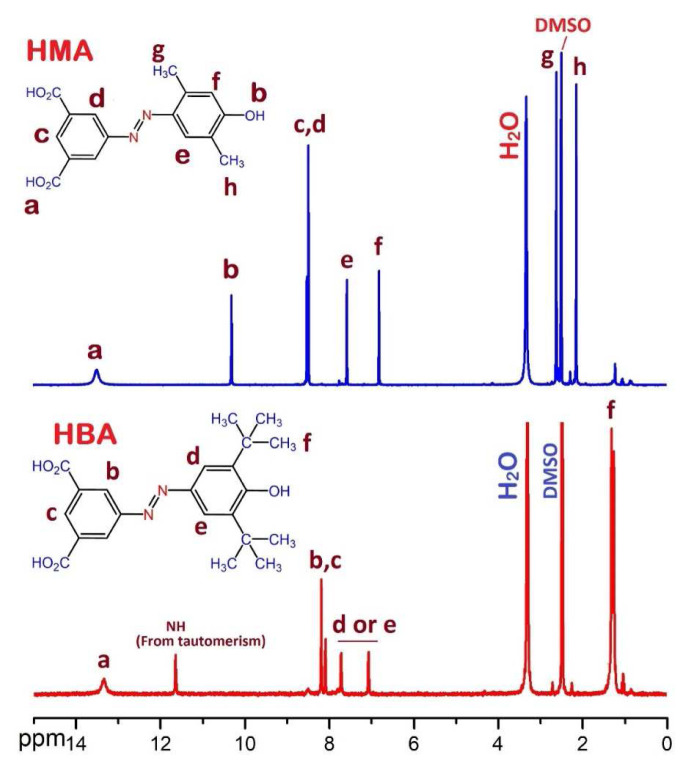
^1^H NMR spectra of HMA and HBA.

The ^1^H NMR spectra of HMA and HBA are depicted in Figure 2. In the ^1^H NMR spectrum of HMA, the singlet signals at 13.49 ppm and 10.29 ppm are assigned to carboxylic acid protons and phenolic OH, respectively. H-aromatics were observed at around 6.80–8.51 ppm. Protons related to two asymmetric methyl groups were observed at 2.56 and 2.13 ppm. In the ^1^H NMR spectrum of HBA, due to the formation of a tautomeric structure no signal for the OH group was observed and the signal related to the NH group was observed at 11.64 ppm instead. In addition, the protons related to the phenolic ring appeared at two individual chemical shifts of 7.06 and 8.09 ppm, which is consistent with previous research [37]. Furthermore, the methyl protons are assigned at 1.22 and 1.26 ppm. All of the above analyses along with ^13^C NMR spectroscopy confirmed the proposed chemical structures of the two synthesized carboxylic acids.

### 3.2. Preparation of PVC nanocomposites

**Scheme 2:**
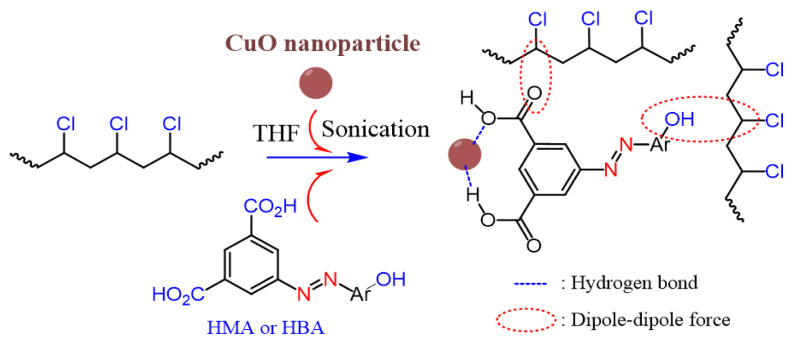
The preparation route for PVC nanocomposite.

The effect of two dicarboxylic acids containing phenol rings as a coupling agent on PVC/CuO nanocomposites’ properties was investigated. It was expected that dicarboxylic acids would act as modifiers of the surface of the CuO nanoparticles during PVC nanocomposite preparation. Dicarboxylic acids are oriented in the nanocomposite so that the carboxyl side is in the direction of CuO nanoparticles; therefore, the other side of the dicarboxylic acids (phenolic ring) is placed on the side of PVC. In the preparation of PVC nanocomposites, the CuO nanoparticles are modified by dicarboxylic acids, which can serve as coupling agents to improve dispersion and interactions between the nanoparticles and the PVC matrix. The hypothesis behind the orientation of dicarboxylic acids in the nanocomposites is based on their chemical structure. Specifically, the carboxyl group (–COOH) of the dicarboxylic acid is thought to interact with the CuO nanoparticles, which have a surface that can accept coordination bonds or hydrogen bonds with the carboxyl group. Meanwhile, the phenolic group of the dicarboxylic acid is oriented toward the PVC matrix, forming interactions such as hydrogen bonding with the PVC chains [18,38], which enhances the compatibility between the filler and the polymer matrix. The schematic preparation route for the PVC nanocomposite is shown in Scheme 2. FT-IR spectroscopy, XRD, and FE-SEM revealed the structure and morphology of PVC sample films. In addition, the heat degradation and mechanical behavior were considered by TGA, Congo red test, and tensile stress–strain test.

#### 3.2.1. FTIR

**Figure 3:**
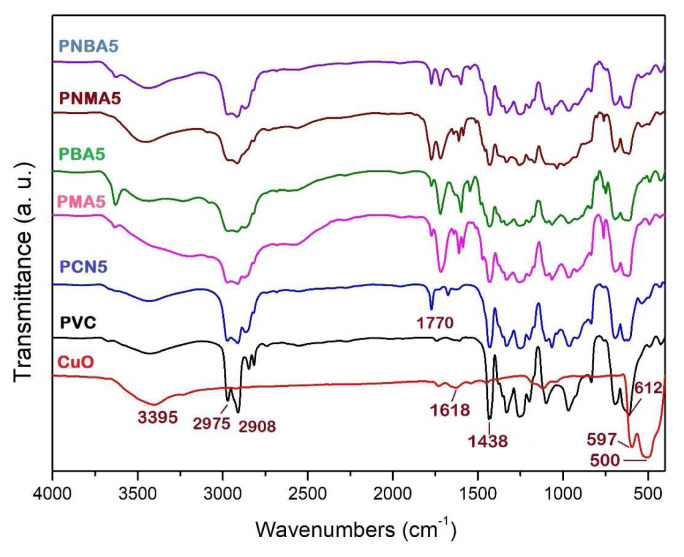
FTIR spectra of CuO and PVC sample films.

The FTIR spectra of the neat PVC, CuO nanoparticles, and the PVC samples are shown in Figure 3. In the neat PVC, the peaks between 2908 and 2975 cm^−1^ and between 1248 and 1438 cm^−1^ correspond to the stretching and vibrational modes of aliphatic CH. The stretching mode of C–Cl is observed at 612 cm^−1^. In the CuO spectrum, the peaks at 500 cm^−1^ and 597 cm^−1^ correspond to the stretching vibrations of Cu–O bonds, confirming the formation of CuO particles. The broad peak centered at 3395 cm^−1^ is attributed to water molecules adsorbed on the surface of the CuO nanoparticles. Additionally, the weak-intensity peak at 1618 cm^−1^ is associated with the bending mode of water molecules [39].

In the spectra of the PVC nanocomposites containing CuO, the peaks between 500 and 600 cm^−1^ are related to the Cu–O bonds, which, due to the low content of CuO, were low intensity. All peaks related to the PVC chain can be observed in the FTIR spectra. In the FTIR spectra of the PMA5, PBA5, and PNBA5 samples, the peaks at around 3620 cm^−1^ are related to the O–H phenolic rings in HMA and HBA structures. Due to the lack of a stabilizer in PVC, the HCl release and carbonyl formation started during PVC film preparation, and absorption bands related to asymmetric and symmetric vibrations related to generated carbonyl groups appeared at around 1724–1770 cm^−1^.

#### 3.2.2. XRD and FE-SEM

**Figure 4:**
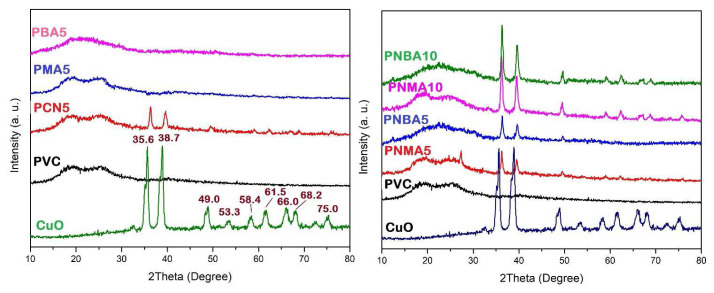
XRD patterns of CuO and PVC sample films.

The XRD analysis of the crystal structure of CuO nanoparticles and PVC samples is depicted in Figure 4. In the CuO pattern, some reflections appeared at 2θ values of 35.6°, 38.7°, 49.0°, 58.4°, 61.5°, 66.0°, 68.2°, and 75.0°, which corresponded to (0 0 2), (1 1 1), (−2 0 2), (2 0 2), (−1 1 3), (−3 1 1), (1 1 3), and (3 1 1) crystallographic planes and revealed a monoclinic crystal structure of CuO nanoparticles with a polycrystalline nature that has been well matched with JCPDS card no. 45-0937 [40]. Furthermore, based on the prominent diffraction peak of CuO at 2θ = 35.63° (corresponding to the (0 0 2) crystallographic plane), the calculated crystallite size using the Debye–Scherrer equation is approximately 21 nm.

In the neat PVC pattern, no sharp reflection was observed, indicating its entirely amorphous structure. Moreover, the XRD patterns of PMA5 and PBA5 samples indicated that the addition of HMA and HBA had no effect on crystallization or change in PVC crystallinity. In the XRD patterns of PVC nanocomposites containing CuO nanoparticles, some reflections related to CuO especially at 2θ values of 35.6° and 38.7° were observed, and their intensities were observed more in samples with higher CuO content such as PNMA10 and PNBA10 as compared to PNMA5 and PNBA5.

**Figure 5:**
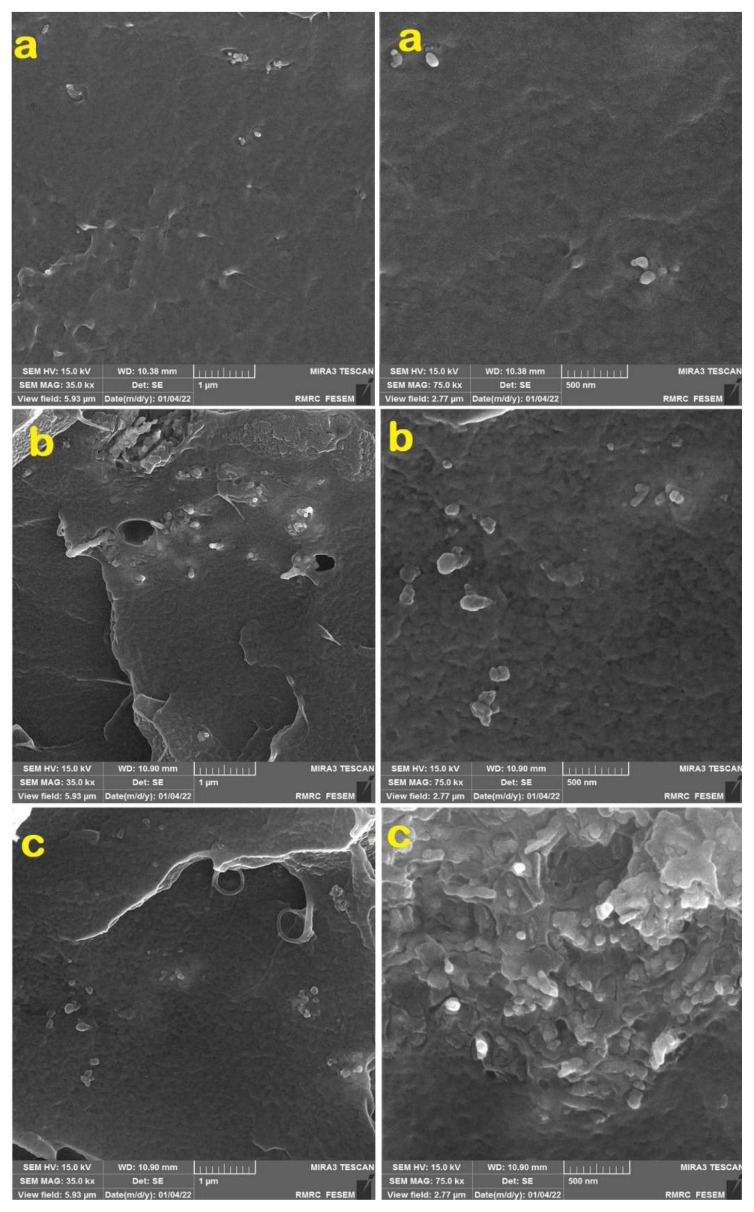
FE-SEM micrographs of a) PCN5, b) PNMA5, and c) PNBA5.

The FE-SEM micrographs of PCN5, PNMA5, and PNBA5 are shown in Figure 5.

The FE-SEM micrographs of the three samples (PCN5, PNMA5, and PNBA5) reveal distinct morphological features that reflect the effects of different additives (CuO, HMA, and HBA) on the dispersion and distribution of CuO nanoparticles within the PVC matrix. The PCN5 sample, which only contains CuO nanoparticles, shows moderate dispersion with visible agglomerates in certain areas. The CuO nanoparticles tend to cluster together, which could be attributed to the strong interaction between the nanoparticles themselves due to their high surface energy. These agglomerates can negatively affect the mechanical and thermal properties of the composite by creating weak points within the polymer matrix. In the PNMA5 sample, where HMA is added alongside CuO nanoparticles, the dispersion improves significantly. The presence of HMA, which contains both carboxyl and phenolic functional groups, likely facilitates better interaction with both the CuO surface and the PVC matrix. The PNBA5 sample, which includes HBA (another hindered phenol derivative), shows the best dispersion of CuO nanoparticles. The morphological differences between these samples highlight the role of the functional groups in improving the interaction between CuO nanoparticles and PVC. The HMA and HBA additives facilitate better dispersion by acting as coupling agents, reducing agglomeration and improving the uniformity of the nanocomposites.

Based on the FE-SEM micrographs, the average particle size of CuO nanoparticles was found to be approximately 25–40 nm (greater than the XRD results due to the organomodification process), consistent with prior studies on CuO nanoparticle dispersion in polymer matrices [41,42]. This particle size from FE-SEM images is larger than that of typical CuO nanoparticles reported in similar systems or XRD results, which is generally due to modification and surrounding by organic compounds and aggregation.

In terms of agglomeration, while the CuO nanoparticles are well dispersed, some slight aggregation can be observed in certain regions of the samples. This could be attributed to the surface energy and the affinity between CuO nanoparticles, which may promote weak interactions in areas with less polymer–nanoparticle interaction. The presence of aggregates may influence the mechanical properties and thermal stability of the nanocomposites. However, the overall dispersion of the nanoparticles, as observed in the FE-SEM images, is significantly better compared to systems in which nanoparticles are not modified [31,43].

**Figure 6:**
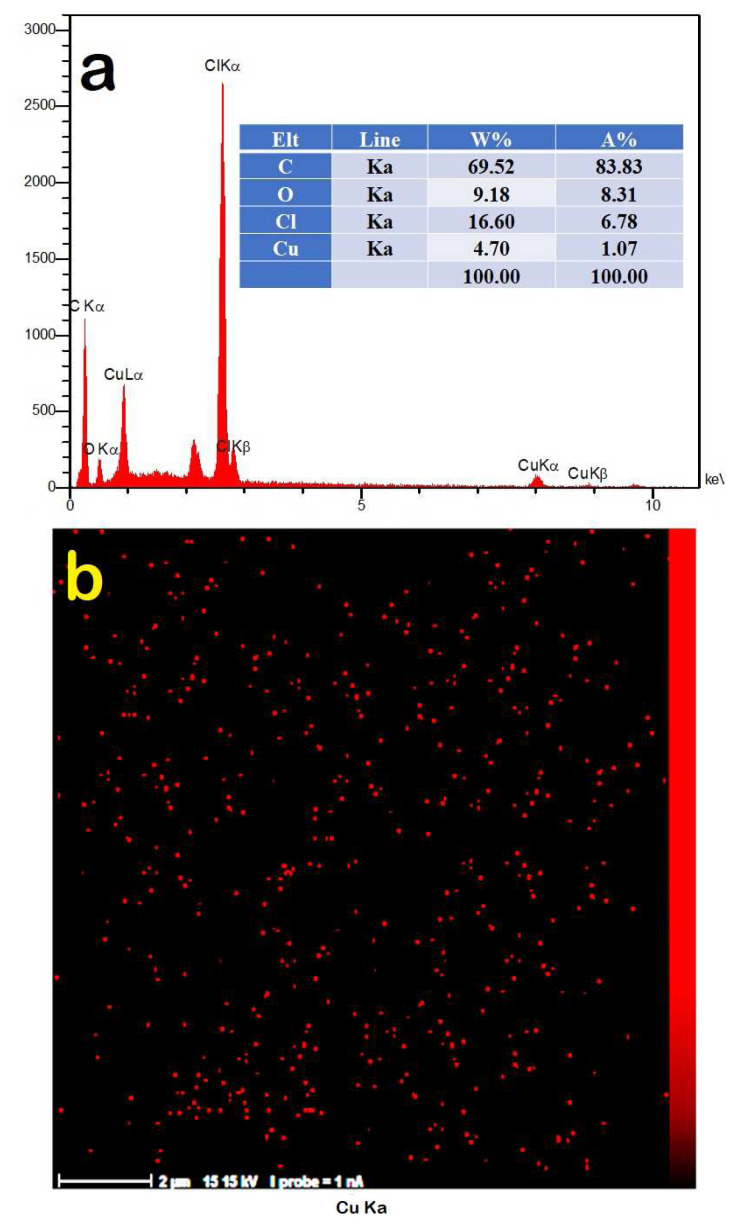
a) EDX spectrum and b) scattering map of Cu element of PNBA5.

The EDX spectrum and elemental distribution map of PNBA5 are shown in Figure 6. The weight percentage of Cu obtained was 4.7, which accommodated with CuO nanoparticle loading in the PVC matrix. Moreover, the Cu elemental mapping image demonstrated a uniform and desirable dispersion of CuO nanoparticles within the PNBA5 nanocomposite.

#### 3.2.3. Thermal properties

**Figure 7:**
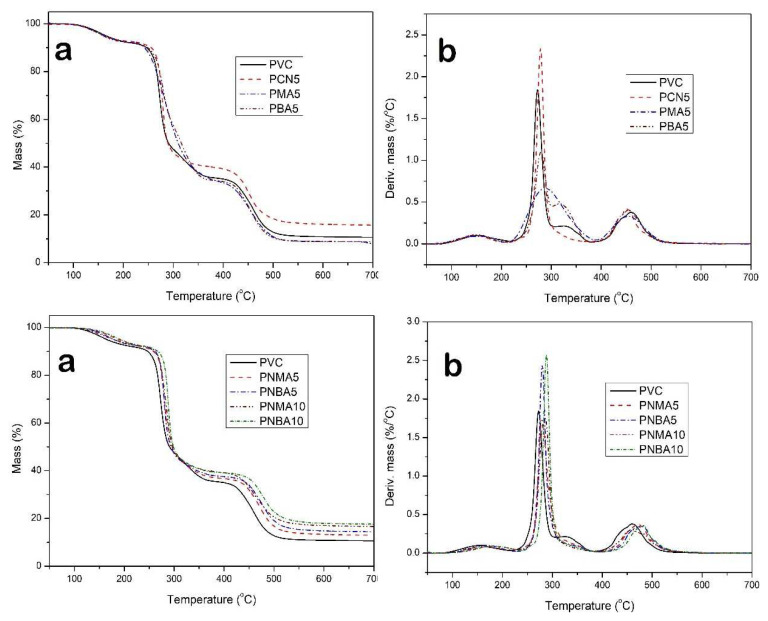
a) TGA and b) DTG curves of the PVC sample films in Ar atmosphere.

**Figure 8:**
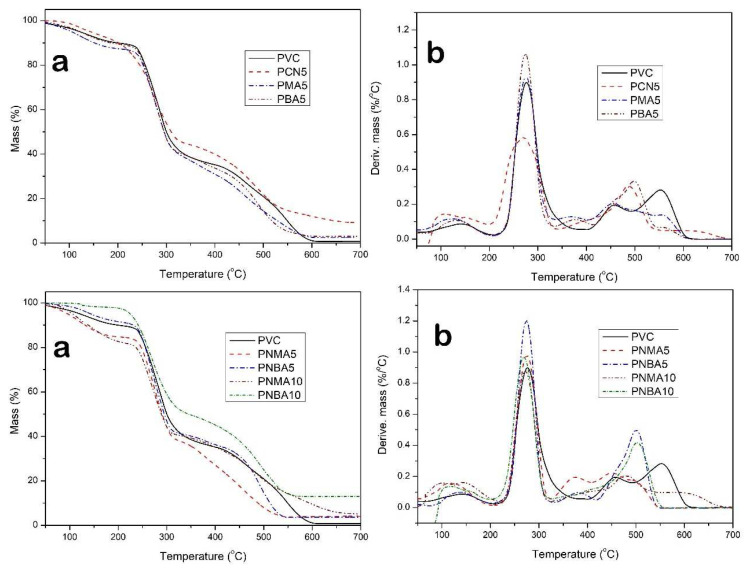
a) TGA and b) DTG curves of PVC sample films in air atmosphere.

TGA of the samples in an inert atmosphere and in the presence of oxygen was used to study the thermal and thermooxidative behavior of the PVC nanocomposites, respectively. The TGA/DTG thermograms of the PVC samples are illustrated in Figures 7 and 8, respectively, and the TGA parameters’ values are given in Table 2.

According to the TGA thermogram of PVC in the Ar atmosphere, three degradation temperature steps were observed, the first of which can be related to the elimination of the remaining solvent and moisture; the second and third stages correspond to the changing structure and degradation of the PVC chain. Regarding the two decomposition steps, it can be explained as follows that the first step, between 260 and 280 °C attributed to the chain degradation, includes the dehydrochlorination process and production of the polyene structure by the deformation of some hydrocarbons such as aromatic structures. This stage is associated with the breaking of the C–Cl bond with the unzipping mechanism, because of having a weak bond with low dissociation energy at 326 kJ mol^−1^. The second step, between 450 and 480 °C, is related to the scission of C–C bonds and the formation of alkyl aromatics through random fragmentation of the PVC chain and a residual char [44]. As shown in Table 2, T_5_, T_10_, and char yield of the neat PVC are observed at 165 °C, 247 °C, and 10%, respectively. By the addition of 5 wt% of CuO nanoparticles (PCN5 sample), T_10_, T_max1_, and char residue were enhanced, while T_5_ and T_max2_ decreased. The decrease in T_5_ may have been due to the increase in residual moisture and solvent with the addition of CuO nanoparticles. The reason for the increase in T_10_ and T_max1_ might be the delay in the diffusion of HCl within the nanocomposite by the formation of CuCl_2_ and trapping of HCl molecules. According to the literature, CuO nanoparticles can promote the transformation of the main structure toward stabilization, which enhances the degree of cross-linking of the PVC chain and decreases the number of broken bonds [45].

By the addition of HMA and HBA without CuO nanoparticles, no significant change in thermal properties was observed, except for T_max1_ in the PMA5 sample, which increased due to the intermolecular forces. By simultaneous loading of phenol derivatives and CuO nanoparticles, the heat resistance of the resulting PVC nanocomposites increased. The maximum thermal stability was observed in the PNBA10 sample, which indicated the presence of CuO nanoparticles along with HBA due to electrostatic forces such as H-bonding and dipole–dipole interactions.

As shown in Table 2, the neat PVC exhibited less initial thermal decomposition and residue values in thermooxidation conditions, as compared to the measurements in the Ar atmosphere. Thermal degradation in the presence of oxygen produces some oxygenated structures such as proxy and hydroperoxides. The thermooxidation results of the nanocomposites exhibited a higher thermal stability for the sample containing HBA, due to the ability to produce more stable radicals.

The maximum thermal resistance was observed for the PNBA10 sample, and its T_5_, T_10_, and char residue values were 224 °C, 241 °C, and 13%, respectively. Moreover, this sample showed the highest T_max2_ of 501 °C. The results exhibited that the simultaneous presence of fillers including CuO nanoparticles and an organic additive containing a hindered phenol structure is necessary to increase the thermal properties of PVC in air, due to the high synergistic effect between their properties. For further study and to clearly explain the above results, the mechanism of thermooxidation of the polymer was discussed as follows.

Unlike thermal degradation in the neutral condition, where the backbone of the polymer and/or at the end of the polymer chain can scission randomly, thermal degradation in the oxidation process occurs via random scissions in the backbone of the polymer, including endothermal and exothermal reactions, and lead to the formation of peroxy radicals and then hydroperoxides that accelerate the thermooxidation processes [46]. Hindered phenol derivatives via the formation of stable radicals during heating and photoirradiation can lead to delayed thermooxidative and photochemical degradation of polymers. In other words, the hindered phenol structure by transformation to stable radicals could act as a radical scavenger during heating and can prevent the activity of radicals formed on the polymer chains.

**Scheme 3:**
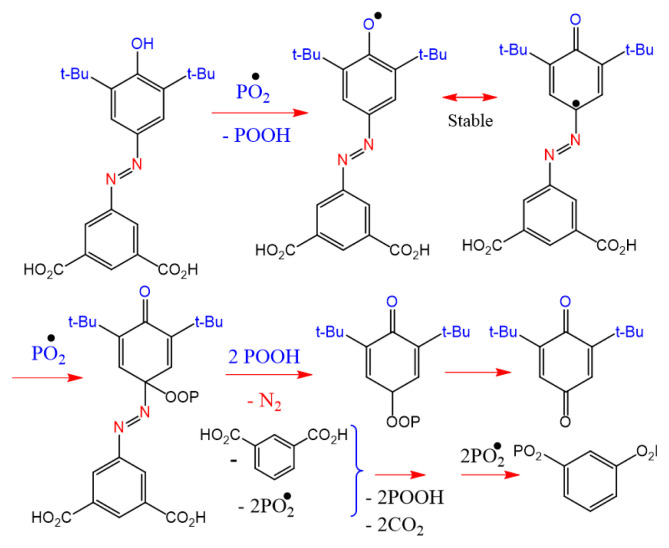
The proposed mechanism for the termination reaction of peroxy radicals.

A proposed mechanism for the termination reaction of HBA with peroxy radicals based on the literature [47] is shown in Scheme 3.

In the presence of oxygen, PVC undergoes oxidative degradation, where the polymer chains are first activated by the formation of peroxy radicals (PO_2_^•^). These radicals can form through direct interaction with oxygen molecules (O_2_) during heating or photoirradiation, which is crucial for the further decomposition of PVC, where the HCl elimination reaction starts, generating active intermediates such as allylic chlorine (Cl^•^), accelerating the degradation process. In the second step, as HCl is released from the PVC backbone, a polyene structure is formed, which is highly conjugated and reactive. In the third step, the hindered phenol derivatives (e.g., HBA) play a significant role as radical scavengers in oxidative conditions. As the peroxy radicals (PO_2_^•^) are generated, they can react with HBA radicals, leading to the formation of stable, nonreactive structures such as quinone derivatives. The interaction between radicals and HBA helps to inhibit further polymer chain degradation by stabilizing the radicals and preventing them from propagating the oxidative degradation process. This scavenging action not only slows down the rate of degradation but also enhances the thermal stability of the polymer matrix. During this process, a 1,3-diradical benzene is also produced, which via reaction with the PO_2_^•^ structure causes more cross-linking of the polymer backbone and, as a result, leads to increased thermal stability.

Furthermore, thermal analysis in air atmosphere for some samples exhibited one more degradation step as compared to thermal analysis in the Ar atmosphere. Except for the first step between 120 and 180 °C, which is related to the removal of remaining THF (as the casting solvent) and moisture, the next stage, which includes 60 wt% mass loss of PVC, can be related to the HCl release, which is the fastest decomposition step, and is quite similar to thermal decomposition in inert gas.

The last decomposition stage, between 370 and 420 °C, which happened at lower temperatures compared to under inert conditions, is directly related to the chain scission. The PVC chains with reduced molecular weight and some gaseous compounds might be generated in the third stage. The generated char decomposed and burned off between 470 and 560 °C, as the fourth degradation stage, along with the most exothermic pyrolysis process [48].

**Figure 9:**
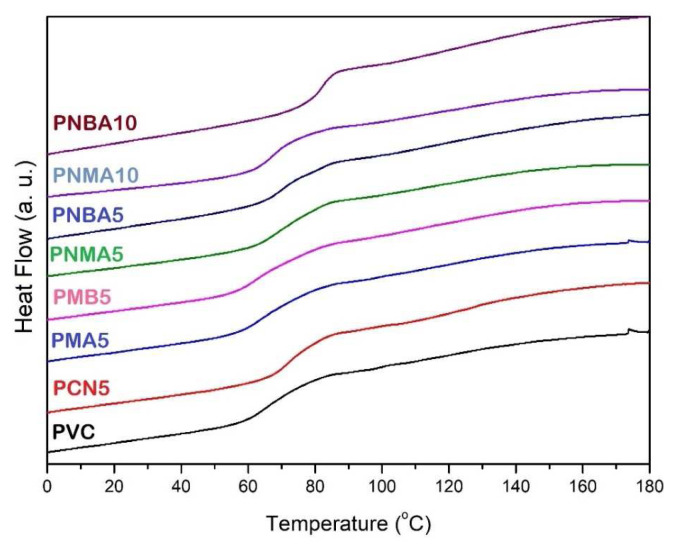
DSC thermograms of the PVC sample films.

The DSC thermograms of the pristine PVC and PVC nanocomposites are presented in Figure 9, and the corresponding glass transition temperatures (T*_g_*) are listed in Table 2. The T_g_ value of the unfilled PVC was 67 °C. The T*_g_* values of two samples, PMA5 and PBA5, were the same as that of the neat PVC, while with 5 wt% loading of CuO nanoparticles into PVC, T*_g_* reached 73 °C. Moreover, the highest T*_g_* was observed for the PNBA10 sample containing 5 wt% of CuO nanoparticles and HBA. The high T*_g_* value can be attributed to various intermolecular interactions, such as hydrogen bonding, which reduce the mobility of the PVC chains.

#### 3.2.4. Congo red test

**Figure 10:**
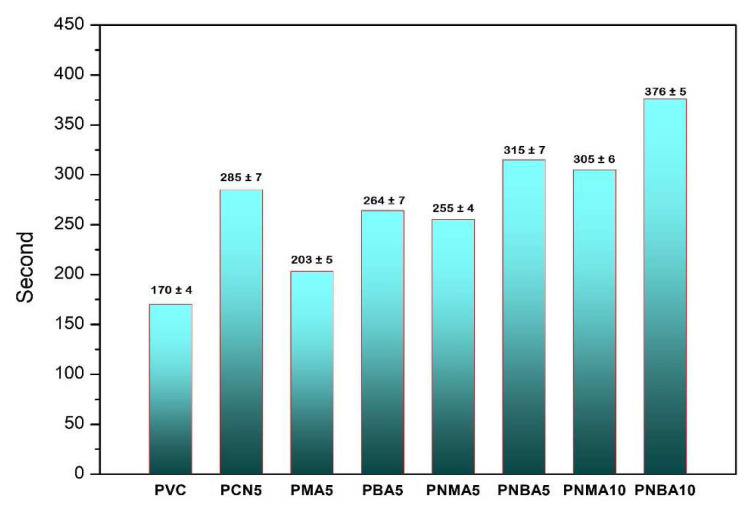
Thermal stability times of the neat PVC and the PVC samples.

The Congo red test is known to be a popular method for the study of the thermal stability and the time to reach dehydrochlorination of PVC composites. In this test, the PVC sample is heated up to 180 °C and the time that the Congo red paper turns blue is considered the thermal stability time. The thermal stability times of the samples are shown in Figure 10.

As seen, the stability time for pristine polymer was 170 s, while by addition of 5 wt% of CuO nanoparticles, it reached 285 s, suggesting that CuO nanoparticles may have reacted with HCl (as mentioned in the TGA section) and led to improved thermal resistance. Furthermore, the addition of HBA to the PVC nanocomposite containing CuO nanoparticles resulted in increased thermal stability times in the PNBA5 and PNBA10 samples. Notably, the longest thermal stability time (376 s) was obtained in the PNBA10 sample, indicating the highest resistance to dehydrochlorination during the pyrolysis of PVC.

According to the proposed mechanisms for the termination reaction of peroxy radicals (Scheme 3), the HBA radicals can scavenge the chlorine radicals and prevent their catalytic effect in the thermal degradation of PVC. Therefore, it is concluded that the PNBA10 sample containing the maximum contents of CuO nanoparticles and HBA exhibits superior resistance to the degradation of PVC, consistent with the TGA/DTG results.

#### 3.2.5. Tensile test

**Figure 11:**
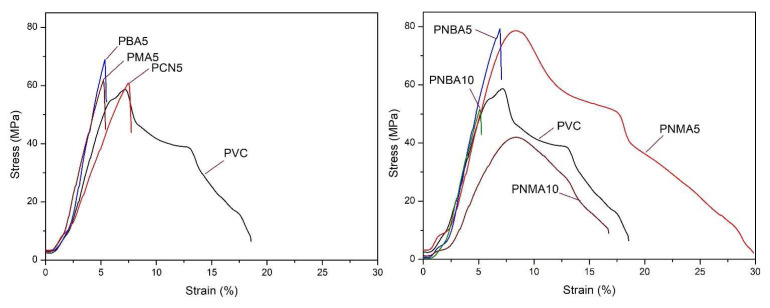
The tensile curves of the PVC sample films.

The effect of the incorporation of the prepared hindered phenol compound and CuO into the PVC matrix on mechanical properties was studied through a tensile test. The tensile stress–strain curves of the samples are depicted in Figure 11 and the corresponding data are listed in Table 3.

The tensile strength of a nanofilled polymer directly depends on the manner of distribution of nanofillers and the compatibility with the main matrix. In general, nanostructured fillers can improve the tensile strength of polymers and the mechanical properties of polymer nanocomposites are influenced by the increasing content of nanofillers [49]. As presented in Table 3, the values for Ts, EB, and YM of the unfilled matrix were 58.99 MPa, 18.49%, and 2.21 GPa, respectively. With CuO nanoparticle loading into the PVC matrix, T_s_ increased to 61.19 MPa, while the EB and YM values decreased to 7.48% and 2.01 GPa, respectively. The decrease in YM can be related to the high surface and rigidity of CuO and these factors also cause decreasing EB values.

By increasing each additive in the PVC matrix up to 2.5 wt%, the tensile strength values in the PNMA5 and PNBA5 samples increased, while increasing additives up to 5 wt% led to a decrease in this parameter. Decreasing tensile strength values with higher loading of additives can be related to the poor distribution of nanoparticles and the formation of the agglomerate area in the nanocomposite structure [50]. However, the higher value of the tensile strength was related to the nanocomposite samples containing HBA.

It is interesting that the highest EB value was obtained (29.93%) in the PMNA5 sample containing 2.5 wt% of each HMA and CuO nanoparticles, almost 10% more than the EB value obtained for the pure PVC. This is despite the fact that the loading of 5 wt% of HMA in the PVC matrix without the presence of CuO nanoparticles caused a decrease in the EB value (PMA5 sample). These results suggest that the simultaneous presence of both CuO nanoparticles and HMA, along with the formation of intermolecular forces with the PVC chains, plays a plasticizing role in the resulting nanocomposite. Furthermore, it is well known that YM is positively correlated with EB according to the Stark–Garton model and that the increasing of YM could lead to reinforced EB of the nanocomposites [51].

The enhanced tensile strength and Young’s modulus of the synthesized PVC/CuO nanocomposites indicate their potential for use in structural and industrial applications requiring robust materials. These include construction components such as wall claddings, floor panels, and roofing materials where mechanical durability under load is essential. The observed elongation at break suggests compatibility with applications demanding flexibility, such as hoses, seals, and cable insulation, where materials must withstand repeated mechanical stress without failure [52]. Furthermore, these improvements directly align with the growing demand for advanced polymer materials in industries seeking lightweight, high-performance, and durable alternatives to traditional materials [53].

## 4. Conclusions

The present study demonstrates the synergistic effect of CuO nanoparticles and hindered phenol derivatives on the mechanical and thermal properties of PVC. The incorporation of these additives significantly enhanced the tensile strength, elongation at break, and thermal stability of PVC, showcasing their potential for industrial applications. The improved properties suggest that the CuO nanoparticles, combined with hindered phenol derivatives, effectively scavenge free radicals and stabilize the polymer matrix during degradation, thereby delaying thermal decomposition and improving overall material performance. The TGA results in an inert atmosphere indicated that the presence of hindered phenol derivative led to an increase in the initial thermal degradation temperature of PVC/CuO nanocomposite. Furthermore, thermooxidation of the PVC samples revealed that the hindered phenol structure with scavenging of free radicals and bridle of peroxides had a significant effect on increasing heat resistance by increasing the onset thermal decomposition and maximum thermal degradation temperatures in the PVC/CuO nanocomposite containing 5 wt% of the hindered phenol derivative. The mechanical test results revealed that the incorporation of 5 wt% each of CuO nanoparticles and HBA into the PVC matrix increased tensile strength compared to pristine PVC.

These findings highlight the potential for tailoring PVC nanocomposites to meet specific application demands, such as in the construction, automotive, and electrical industries, where mechanical durability and thermal resistance are critical. Moreover, the study underscores the importance of additive selection and dispersion strategies in optimizing polymer performance. Future work could focus on exploring other metal oxide nanoparticles or synergistic combinations to further enhance these properties and expand the application scope of PVC-based materials.

## Figures and Tables

**Table 1:** Formulations of the PVC nanocomposites.

Sample	PVC^a^ (wt%)	CuO^b^ (wt%)	HMA^c^ (wt%)	HBA^d^ (wt%)

PVC	100	0	0	0
PCN5	95	5	0	0
PMA5	95	0	5	0
PBA5	95	0	0	5
PNMA5	95	2.5	2.5	0
PNBA5	95	2.5	0	2.5
PNMA10	90	5	5	0
PNBA10	90	5	0	5

a Neat PVC,

b CuO nanoparticles,

c 5-((4-hydroxy-2,5-dimethylphenyl)diazenyl)isophthalic acid,

d 5-((4-hydroxy-3,5-di-tert-butylphenyl)diazenyl)isophthalic acid.

**Table 2:** Thermal properties of the PVC films in Ar and air atmospheres.

Sample code	Thermal properties in Ar						Thermal properties in air						T*_g_*^f^

	T_5_^a^	T_10_^b^	CY^c^	T_max1_^d^	T_max2_	T_max3_	T_5_	T_10_	CY	T_max1_	T_max2_	T_max3_	
PVC	165	247	10	272	460	-e	122	199	<1	276	455	552	67
PCN5	160	256	16	280	451	-	144	201	10	271	486	-	73
PMA5	163	242	8	288	452	-	114	151	2	274	455	556	66
PBA5	159	248	9	280	455	-	122	185	3	272	499	-	66
PNMA5	188	258	13	279	470	-	96	131	4	276	374	470	71
PNBA5	179	262	14	278	479	-	144	231	4	276	382	497	71
PNMA10	178	260	17	288	467	-	105	138	5	272	480	-	72
PNBA10	192	267	18	288	475		224	241	13	268	501	-	81

a,b Temperature at 5% and 10% mass loss (°C);

c Char yield (CY) (%), weight percentage of material remaining after TGA analysis at a maximum temperature of 700 °C;

d Main mass loss temperature (°C);

e Not observed;

f Glass transition temperature (T*_g_*) recorded by DSC at a heating rate of 10 °C/min in an argon atmosphere (°C).

**Table 3:** Mechanical properties of the PVC films.

Sample code	T*_s_* (MPa)^a^	EB (%)^b^	YM (GPa)^c^
PVC	58.99 ± 1.22	18.49 ± 1.04	2.21 ± 0.07
PCN5	61.19 ± 2.31	7.48 ± 1.51	2.01 ± 0.13
PMA5	68.97± 1.98	5.35 ± 0.96	3.02 ± 0.09
PBA5	62.12 ± 3.14	6.78 ± 1.39	2.89 ± 0.17
PNMA5	78.65 ± 2.28	29.93 ± 2.41	2.36 ± 0.12
PNBA5	79.51 ± 2.03	6.94 ± 1.34	2.50 ± 0.13
PNMA10	42.08 ± 2.61	16.67 ± 1.84	1.35 ± 0.15
PNBA10	51.50 ± 1.74	5.10 ± 1.19	2.36 ± 0.12

a Tensile strength,

b Elongation at break,

c Young’s modulus.
